# Effects of incentive spirometry on cardiopulmonary parameters, functional capacity and glycemic control in patients with Type 2 diabetes

**DOI:** 10.1142/S1013702520500110

**Published:** 2020-04-30

**Authors:** Happiness Anulika Aweto, Esther Onyinyechi Obikeh, Bosede Abidemi Tella

**Affiliations:** 1 Department of Physiotherapy, Faculty of Clinical Sciences, College of Medicine, University of Lagos, PMB 12003, Idi-Araba, Lagos, Nigeria; awetohappiness@gmail.com; haweto@unilag.edu.ng

**Keywords:** Type 2 diabetes mellitus, incentive spirometry, cardiopulmonary function, functional capacity, glycemic control

## Abstract

**Background:**

Patients with Type 2 diabetes mellitus (T2DM) suffer cardiopulmonary impairment and may present with weakness of the inspiratory muscles.

**Objective:**

This study was designed to determine the effects of incentive spirometry (IS) on selected cardiopulmonary parameters, functional capacity and glycemic control in patients with T2DM.

**Methods:**

Fifty-nine participants (25 males and 34 females) recruited from the out-patient clinic of the Department of Medicine of two hospitals in Lagos State, Nigeria, who were randomly assigned into two groups, completed the study. In addition to the medical management of T2DM, IS group received incentive spirometry while control group continued with the medical management of T2DM alone. Selected cardiovascular parameters, pulmonary parameters, functional capacity (using 6-min walk test) and fasting blood glucose level were assessed at baseline and at the end of eight weeks intervention period. Data were analyzed using the Statistical Package for Social Sciences (SPSS Version 21). Level of significance was set at p<0.05.

**Results:**

There were statistically significant improvements in all the cardiovascular parameters (p=0.001) of IS group except systolic blood pressure. There were significant changes in all the pulmonary parameters, functional capacity and glycemic control (p<0.05) of IS group while there was none in control group. There were significant differences between the mean changes of various selected outcome measures of the two groups (p<0.05) except for diastolic blood pressure and blood glucose level.

**Conclusion:**

IS had positive effects in improving cardiopulmonary function, functional capacity and glycemic control in patients with T2DM.

## Introduction

Diabetes is a group of metabolic diseases in which a person cannot regulate the amount of sugar, specifically glucose, in the blood.^1^ This high blood sugar produces the classical symptoms of polyuria, polydipsia and polyphagia.^2^ Type 2 diabetes mellitus (T2DM) is the most common type of diabetes, it accounts for about 90–95% of all cases of diabetes mellitus, and therefore of primary interest.^3^ It is characterized by insulin resistance which may be combined with relatively reduced insulin secretion.^4^ It can lead to glycation of tissues, which proceeds with acute metabolic disturbances and ends with organ damage with severe health deteriorations.^5^ The International Diabetes Federation (IDF) estimated that in 2011, 366 million people (8.3% of the world population) had diabetes worldwide, a figure expected to reach 552 million (9.9% of the world population) by the year 2030, with 80% of people with diabetes living in low and middle-income countries. Worldwide, approximately 200 million people have T2DM.^6^

Diabetes is known to have a profound impact on life expectancy; it is also known to affect a patient’s general health and well-being. It is a significant health problem with microangiopathy and macroangiopathy resulting in many complications like, diabetic neuropathy, diabetic retinopathy, diabetic nephropathy, peripheral vascular disease, cardiovascular disease, susceptibility to infections and periodontal disease.^7,8,9,10^ The presence of an extensive microvascular circulation and abundant connective tissue in the lungs raises the possibility that lung tissue may be affected by microangiopathy process and non-enzymatic glycosylation of tissue proteins, induced by chronic hyperglycemia renders the lung a “target organ” in patients with T2DM. Since normal lung mechanics and gas exchange are influenced by the integrity of pulmonary connective tissue and microvasculature, abnormalities in either of these two structural components of the lung may lead to the development of measurable abnormalities of pulmonary function.^11^

Hyperglycemia-induced microangiopathy via increased oxidative stress has also been shown to impair pulmonary and cardiovascular function.^12^ Vaibhav and Chahar^9^ posit that the pulmonary function abnormalities reported in T2DM patients is usually associated with chronic hyperglycemia. Deterioration of pulmonary function is seen in 80% of all patients with T2DM from the entire world population.^11^ The relationship between T2DM and pulmonary function remains important because of potential epidemiological and clinical implications.^13^ In 2004, Davis *et al.*^14^ observed that measures of airflow limitation predict all-cause mortality in diabetes and intensive glycemic management may reduce the risk of death through improved ventilatory function independent of other beneficial effects. The function of inspiratory muscles is also frequently found to be impaired (decreased strength and/or endurance) in patients with T2DM.^15^ This leads to poor exercise tolerance and decreased functional capacity.^4^ So, the assessment of pulmonary function is an important investigation because early detection of pulmonary function impairment and its appropriate treatment will help to reduce morbidity and mortality. For these reasons, specific inspiratory muscle training (IMT) could be justified as a strategy with potential clinical benefits in patients with T2DM.^8^ IMT can be achieved by using an inspiratory muscle trainer or incentive spirometry (IS).^16,17^

Incentive spirometry is frequently used and recommended for reducing the chance of pulmonary complications and improving pulmonary function in patients with COPD, but its efficacy in the improvement of pulmonary function in patients with T2DM is still uncertain.^17,18^ Incentive spirometers are affordable and easy to use, have no side effect, and provide a direct feedback that act as an incentive for exercise.^19^ Despite such advantages, the use of incentive spirometers to improve the endurance of respiratory muscles and pulmonary function indices of patients with T2DM is not a typical practice, and there is little evidence supporting their efficacy for this application.^17^ Since there is a relationship between the cardiopulmonary function and functional capacity as well as glycemic levels in patients with T2DM, any intervention that affects the respiratory function may in turn affect the other parameters.^20,21^ This study was therefore designed to determine the effects of IS on selected cardiopulmonary parameters, functional capacity and glycemic control in patients with T2DM and the correlation between pulmonary function and functional capacity, duration of the disease (DOD) and glycemic control.

## Methods

This study was a single blinded randomized controlled trial.

### Subjects

A total of 70 patients diagnosed with T2DM were recruited for this study from the out-patient clinic of the Department of Medicine, Lagos University Teaching Hospital (LUTH), Idi Araba, Lagos State, Nigeria and General Hospital Lagos (GH), Marina, Lagos State, Nigeria. The participants were recruited based on the following inclusion criteria: Confirmed medical diagnosis of T2DM, patients above 18 years of age who had the ability to follow instructions and engage in study procedures, no structured aerobic exercise program in the preceding six months. They were non-smokers and non-alcoholics with no physical restriction in terms of mobility and had no pre-existing pulmonary infections or deformities in the trunk. Patients who have had recent abdominal surgery and those with severe complications of DM, such as neuropathy, nephropathy, and retinopathy, were excluded from the study. The sample size was determined using this formula: n=N ($Z_{1}+Z_{2}{)}^{2}$/ ES^2^. Where n=minimum sample size, N=number of groups, $Z_{1}=\alpha$ confidence of interval at 0.05=1.96, $Z_{2}=\beta$ confidence of interval at 0.20=0.84, ES=Effect size=0.80 (using Cohen’s standard effect size).^22^ The number of participants for both groups was calculated as 30, this means that the calculated minimum sample size for each study group was 30∕2=15. Of the 70 patients recruited, six were excluded from this study. The remaining 64 were randomly assigned into two different groups (IS and Control) with 32 patients in each group using fish bowl technique. In this sampling technique, each unit of the population is represented by a slip of paper containing a number, the slips of paper are put in a box and shuffled, and the slips are then pulled out one by one without looking at them, until the number of slips selected equals the sample size.^23^ The IS group received medical management of T2DM and IS while the control group continued with the medical management of T2DM alone.

However, 59 participants (25 males and 34 females) completed the study with five withdrawals from the study; two from the IS group could not keep up with the weekly appointments and three from the control group did not show up for the post test assessment. In the IS group, 14 males and 16 females completed the study while in control group 11 males and 18 females completed the study.

Ethical approval was obtained from the Research and Ethics Committee of LUTH (ADM/DCST/HREC/APP/1538) and GH Lagos through the Lagos State Health Service Commission (HSC) (LSHSC/2222/XXIII/533). All participants gave written informed consents to participate in this study. This clinical trial was registered by the Pan African Clinical Trial Registry on the 16th of November, 2017 with an identification number PACTR201711002754229.

### Instruments

The following instruments were used for this study:

A flow-sensing triflo II incentive spirometer (India model SG523130993), Spirometer (Contec SP 10, China, Version 1.2), Mercury sphygmomanometer (Accoson UK, BS EN 1060-1), Stethoscope (Littmann Classic II SE, UK), Glucometer (Accu-Chek Active, Germany), Stadiometer (Seca 213, UK), Portable bathroom weighing scale (Camry BR9312, China), 30-meter wind-up tape measure (Starrett, USA), Stopwatch (Nivia JS 307, India), Mechanical lap counter (HDE, USA), Two small cones and a plastic chair with back rest.

### Pre-intervention assessment

On arrival at the study site, each participant was formally welcomed and seated on a chair with back rest located near the starting position of the 30 m walking course for 10 min before the commencement of tests. During this time, the participants were examined for possible contra-indications after which the purpose and protocol for the study were adequately explained to each of them before seeking and obtaining their written informed consent using a consent form. All participants who gave their consent to participate in the study had their baseline measurements of heart rate, blood pressure (using a stethoscope and sphygmomanometer), lung function (using a spirometer), blood sugar level (using a glucometer) and functional capacity (using 6 min walk test (MWT)) assessed and recorded. Socio-demographic data and physical characteristics such as; age, gender, height, weight and body mass index were also obtained from the participants.

Blood sugar level was measured according to the American Diabetes Association Criteria.^24^ Cardiovascular parameters of heart rate, systolic and diastolic blood pressure (SBP and DBP) were measured using a sphygmomanometer and a stethoscope. Rate pressure product was calculated as the product of heart rate and systolic blood pressure.^7,25^

Pulmonary function was assessed with each of the participant seated comfortably in a chair with back rest, feet firmly on the ground and all constricting clothing such as braziers and waist belts loosened to prevent alteration of test results from restricted thoracic expansion and abdominal mobility. Guidelines suggested by American Thoracic Society/European Respiratory Society (ATS/ERS) were adopted during this technique.^26^ The spirometer was cleaned with an alcohol wipe and disposable mouthpiece was used for each participant. The participants were instructed to breathe in as deeply as possible (full inspiration) and hold their breath just long enough to seal their lips around the mouthpiece and to clip the nose with a nose clip. They are then instructed to blow out through the mouth (exhale) into the mouthpiece forcibly, as hard, as fast and as long as possible (full expiration), until there is no air left to expel (at least for 6 s).^27^ The procedure was repeated thrice at 15 min interval and the forced vital capacity (FVC), forced expiratory volume in 1 s (FEV${}_{1})$ and peak expiratory flow rate (PEFR) readings were obtained.^25^ It was ensured that repeatability was considered to be adequate, for the FVC and FEV_1_, when the two highest values were within 0.150 L of each other and the higher value between the two repeatable values was the accepted value. The highest value of PEFR was the accepted value.^27,28^

Functional capacity was assessed using the 6MWT which is a performance-based tool. Prior to commencement of the test, participants were informed that the objective of the test was to walk as far as possible for 6 min by walking back and forth around the cones as quickly as possible but not to run or jog. This was demonstrated to the participants by walking one lap. Standardized instructions and words of encouragement were given to the participants during the test as provided by the ATS^29^ guidelines for the 6MWT. The total distance covered by each participant in 6 min was calculated by multiplying the number of laps walked by 60 m (one lap is to and fro the 30 m walk course) plus the final partial lap in meters.^29^

### Intervention

In addition to the medical management of their condition, participants in the IS group were taught how to use the flow-sensing incentive spirometer following a standard testing protocol as suggested by the American Association for Respiratory Care (AARC).^18,30^ The participants were seated comfortably in an upright position and instructed to hold the incentive spirometer in an upright position, exhale normally, and then place the lips tightly around the mouthpiece. The next step was a slow inhalation to raise the ball (flow-oriented) in the chamber. At maximum inhalation the mouthpiece was removed, followed by a breath-hold (sustained for a minimum of 3 s), and normal exhalation. Participants were advised to always assume an upright position during the IMT as inspiratory muscle function is optimized in the upright position while recumbent or semi-recumbent postures impair respiratory muscle function.

Inspiratory spirometry was performed two days in a week at the clinic and the participants were encouraged to carry out training at home every day at the frequency of 10 breaths per session, five times a day for eight weeks.^31^ On the other hand, participants in the control group continued receiving standard medical management without any further intervention. Participants kept daily logs of there IS sessions indicating the date and time of their training sessions. Also, all participants’ phone numbers were obtained for individual home sessions and follow up. Participants withheld any structured aerobic exercise program during the experimental period.

### Post-intervention assessment

The outcome variables were assessed on the next day at the end of the eighth week of study for the participants who completed their individual group assignment using the same procedure as pre-intervention assessment.

## Data Analysis

Data was analysed using the SPSS Version 21. Data was summarized using descriptive statistics of frequency, mean and standard deviation. Independent t-test was used to analyze the data between the groups (IS and control) and paired t-test was used to analyze the outcomes within the two groups. Pearson correlation was used to determine the relationship between pulmonary function and functional capacity, glycemic control as well as DOD. The level of significance was set at p<0.05.

**Table 1.** Comparison of baseline physical and other characteristics of both groups.

	Mean ± SD				
Variables	IS group (n=30)	Control group (n=29)	All participants (n=59)	t-value	p-value
Age (years)	54.47 ± 9.77	55.76 ± 14.56	55.10 ± 12.64	−0.399	0.692
Weight (Kg)	71.97 ± 11.23	71.76 ± 10.26	71.86 ± 10.67	0.074	0.941
Height (m)	1.69 ± 0.09	1.66 ± 0.07	1.67 ± 0.08	1.171	0.247
BMI (Kg/m${}^{2})$	25.50 ± 4.63	26.11 ± 4.07	25.80 ± 4.34	−0.532	0.597
DOD (years)	10.53 ± 6.77	6.66 ± 4.89	8.63 ± 6.19	2.529	0.014*

*Notes*: * Significant at p<0.05.

BMI: Body mass index; DOD: Duration of disease; SD: Standard deviation; n: Number of participants in a group; Kg: Kilogram; m: meters.

**Table 2.** Comparison of baseline variables of both groups.

	Mean ± SD			
Variables	IS group	Control group	t-value	p-value
FVC (L)	2.02 ± 0.61	1.98 ± 0.51	0.307	0.760
FEV_1_(L)	1.80 ± 0.56	1.67 ± 0.53	0.947	0.347
PEFR (L/s)	3.43 ± 1.39	4.02 ± 1.84	−1.367	0.177
SBP (mmHg)	134.13 ± 15.66	135.72 ± 15.73	−0.389	0.699
DBP (mmHg)	89.20 ± 6.94	83.83 ± 7.96	2.335	0.024*
HR (bpm)	74.90 ± 9.10	75.83 ± 11.56	−0.342	0.734
RPP	10017.73 ± 1487.02	10318.97 ± 2105.57	−0.633	0.530
FBS (mg/dl)	121.13 ± 32.81	121.86 ± 43.42	−0.073	0.942
6MWD (m)	200.77 ± 43.39	166.31 ± 62.09	2.463	0.017*

*Notes*: *Significant at p<0.05.

FVC: Forced vital capacity; FEV_1_: Forced expiratory volume in 1 s; PEFR: Peak expiratory flow rate; SBP: Systolic blood pressure; DBP: Diastolic blood pressure; HR: Heart rate; RPP: Rate pressure product; FBS: Fasting blood sugar; 6MWD: 6 min walk distance.

## Results

The mean age, BMI and DOD of the participants in the IS and control groups were 54.47±9.77 years and 55.76±14.56 years, 25.50±4.63 kg/m^2^ and 26.11±4.07 kg/m^2^ as well as 10.53±6.77 years and 6.66±4.89 years, respectively. There was no significant difference in the physical and other baseline characteristics of the two groups except DOD (p=0.014) (Table 1). At baseline, there was no significant difference in the cardiopulmonary function parameters of the participants in the two groups with the exception of DBP which showed a significant difference (p=0.024). The 6-min walk distance (6MWD) of the participants in the two groups also showed a significant difference at baseline (p=0.017) while FBS showed no significant difference (Table 2).

**Table 3.** Comparison of the pre- and post-mean values of the pulmonary parameters, cardiovascular parameters, FBS and 6MWD in IS group.

	Mean ± SD			
Variables	Pre	Post	t-value	p-value
FVC (L)	2.02 ± 0.61	2.50 ± 0.76	−4.586	0.001*
FEV_1_ (L)	1.80 ± 0.56	2.16 ± 0.67	−5.134	0.001*
PEFR (L/s)	3.43 ± 1.39	4.85 ± 1.62	−7.621	0.001*
SBP (mmHg)	134.13 ± 15.66	130.40 ± 17.20	1.587	0.123
DBP (mmHg)	89.20 ± 6.94	82.60 ± 9.91	4.902	0.001*
HR (b/m)	74.90 ± 9.10	68.97 ± 11.54	4.179	0.001*
RPP	10017.73 ± 1487.02	8966.40 ± 1786.79	4.282	0.001*
FBS(mg/dl)	121.13 ± 32.81	102.13 ± 36.90	2.804	0.009*
6MWD (m)	200.77 ± 43.39	229.50 ± 53.32	−6.845	0.001*

*Notes*: * Significant at p<0.05.

FVC: Forced vital capacity; FEV_1_: Forced expiratory volume in 1 s; PEFR: Peak expiratory flow rate; SBP: Systolic blood pressure; DBP: Diastolic blood pressure; HR: Heart rate; RPP: Rate pressure product; FBS: Fasting blood sugar; 6MWD: 6 min walk distance.

**Table 4.** Comparison of the pre- and post-mean values of the pulmonary parameters, cardiovascular parameters, FBS and 6MWD in the control group.

	Mean ± SD			
Variables	Pre	Post	t-value	p-value
FVC (L)	1.98 ± 0.51	1.98 ± 0.52	−0.095	0.925
FEV_1_ (L)	1.67 ± 0.53	1.69 ± 0.51	−0.928	0.361
PEFR (L/s)	4.02 ± 1.84	4.07 ± 1.84	−0.506	0.616
SBP (mmHg)	135.72 ± 15.73	135.17 ± 12.95	0.510	0.614
DBP (mmHg)	83.83 ± 7.96	83.83 ± 7.96	−0.225	0.824
HR (b/m)	75.83 ± 11.56	76.72 ± 8.73	−0.958	0.346
RPP	10318.97 ± 2105.57	10373.90 ± 1543.84	−0.326	0.747
FBS (mg/dl)	121.86 ± 43.42	114.93 ± 28.53	1.547	0.133
6MWD (m)	166.31 ± 62.09	173.75 ± 58.99	−1.824	0.079

*Notes*: * Significant at p<0.05.

FVC: Forced vital capacity; FEV_1_: Forced expiratory volume in 1 s; PEFR: Peak expiratory flow rate; SBP: Systolic blood pressure; DBP: Diastolic blood pressure; HR: Heart rate; RPP: Rate pressure product; FBS: Fasting blood sugar; 6MWD: 6 min walk distance.

Tables 3 and 4 show the comparisons of pre and post intervention mean values of the pulmonary parameters, cardiovascular parameters, fasting blood sugar level (FBS) and functional capacity (6MWD) in the IS group and control group, respectively. The paired t-test analysis showed statistically significant improvements in all the pulmonary function parameters, FBS and 6MWD in the IS group (p<0.05). There were also significant improvements in all cardiovascular parameters in the IS group (p<0.05) except for SBP which showed no significant difference (p=0.123) (Table 3).

There were no significant differences in any of the pulmonary function parameters, cardiovascular parameters, FBS and 6MWD in control group (p>0.05) (Table 4).

The comparison of the mean changes in the pulmonary parameters, cardiovascular parameters, FBS and 6MWD post intervention (at the end of the eighth week) between the two groups showed significant differences in all the pulmonary function parameters (FVC, FEV_1_ and PEFR) (p=0.001, respectively) and 6MWD (p=0.002). There were also significant differences in all the cardiovascular parameters (SBP, HR and RPP) (p<0.05) except for DBP. No significant difference was seen between the mean changes in FBS of the two groups (Table 5).

**Table 5.** Comparison of the mean changes in the pulmonary parameters, cardiovascular parameters, FBS and 6MWD Post-intervention between the two groups.

	Mean changes ± SD			
Variables	IS Group	Control Group	t-value	p-value
FVC	0.48	0.02	5.041	0.001*
FEV_1_	0.36	0.02	4.697	0.001*
PEFR	1.42	0.05	4.933	0.001*
SBP	3.73	0.55	3.356	0.002*
DBP	6.60	0.01	1.784	0.080
HR	5.93	0.89	2.854	0.006*
RPP	1051.33	54.93	3.569	0.001*
FBS	19.00	6.93	1.572	0.123
6MWD	28.73	7.44	3.255	0.002*

*Notes*: *Significant at p<0.05.

FVC: Forced vital capacity; FEV_1_: Forced expiratory volume in 1 s; PEFR: Peak expiratory flow rate; SBP: Systolic blood pressure; DBP: Diastolic blood pressure; HR: Heart rate; RPP: Rate pressure product; FBS: Fasting blood sugar; 6MWD: 6 min walk distance.

**Table 6.** Relationship between changes in pulmonary function and functional capacity; glycemic control; duration of the disease of the participants.

Variables	FVC	FEV_1_	PEFR
6MWD	r=0.176	r=0.250	r=0.281
	p=0.186	p=0.058	p=0.033*
FBS	r=0.029	r=0.181	r=0.141
	p=0.830	p=0.171	p=0.286
DOD	r=0.220	r=0.204	r=0.333
	p=0.085	p=0.122	p=0.010*

*Notes*: *Significant at p<0.05.

FVC: Forced vital capacity; FEV_1_: Forced expiratory volume in 1 s; PEFR: Peak expiratory flow rate; FBS: Fasting blood sugar; 6MWD: 6 min walk distance; DOD: Duration of disease; r: Correlation coefficient; p: Significance value.

Correlation Guide: 0.00–0.19 “Very weak”; 0.20–0.39 “Weak”; 0.40–0.59 “Moderate”; 0.60–0.79 “strong”; 0.80–1.0 “Very strong”; (Evans *et al.* 1996).

The analysis of the relationship between changes in pulmonary function (FVC, FEV_1_ and PEFR) and 6MWD, FBS as well as the DOD of the participants using the Pearson’s correlation coefficient showed that there was no correlation between 6MWD, FBS, DOD and FVC, FEV_1_. There was a significant correlation between PEFR and 6MWD (r=0.281;p=0.033) as well as DOD (r=0.333;p=0.010) (Table 6).

## Discussion

The purpose of this study was to determine the effects of IS on selected cardiovascular parameters, pulmonary parameters, functional capacity and glycemic control in patients with T2DM and the correlation between pulmonary function and functional capacity, DOD and glycemic control. IS had significant effect on all cardiovascular parameters (DBP, HR and RPP) except SBP. It had significant effect on all pulmonary parameters (FVC, FEV_1_ and PEFR), functional capacity and glycemic control. There was no significant correlation between the changes in pulmonary function (FVC and FEV${}_{1})$ and functional capacity, glycemic control as well as DOD except weak correlation between PEFR and functional capacity as well as DOD.

The significant different baseline data in DOD, DBP and 6MWD between the two groups (Tables 1 and 2) did not matter after all as the mean changes of these outcome variables were eventually used for comparison between the two groups (Tables 5 and 6) instead of the actual values post intervention. Among these three parameters, only DBP showed no statistically significant difference when the mean changes of the selected outcome measures were compared between the two groups (Table 5) even though the mean changes in DBP was more in the IS group (6.60 mmHg) than the control group (0.01 mmHg).

The finding that there were significant changes in pulmonary function parameters (FVC, FEV_1_ and PEFR) of participants in the IS group following eight weeks of IS using incentive spirometer while there was no significant change in the control group implies that IS training brought about the change. This is also buttressed by the significant differences observed in the comparison between the mean changes in pulmonary function parameters of the two groups. This improvement could be associated with the fact that the visual feedback provided by the incentive spirometer encourages the inspiratory performance of the participants and sustained maximal inspiration and hyperpnoea aim to gain pulmonary volume.^32^ It is also known that maximum inspiration causes the increase of the trans-pulmonary pressure and the increase of the pulmonary volume. Furthermore, resting at the end of inspiration keeps up the increase of the trans-pulmonary pressure and ensures the alveolar stability. All of these enhance gas exchange, improve lung compliance, perfusion and reduce the work of breathing.^33^

This corroborates the findings of Paiva *et al.*^16^ who investigated the effect of IMT in healthy sedentary females and reported an increase in pulmonary function post IMT using incentive spirometer for 30 days. However, these findings differ from that of Correa *et al.*^4^ which showed no significant change in the pulmonary function of patients with T2DM with inspiratory muscle weakness who were grouped into an experimental and a placebo group. The experimental group had eight weeks of IMT and the authors explained that this result could be because diabetes mellitus results in attenuation of inspiratory muscle metaboreflex.^4^ Increase in inspiratory muscle strength with no consequent increase in pulmonary function has also been documented for patients with diabetic autonomic neuropathy post IMT.^15^ This difference could be because of the small sample size used in these studies. Likewise, Bavarsad *et al.*^34^ reported no significant change in pulmonary function of patients with chronic obstructive pulmonary disease (COPD) who had eight weeks of IMT using a flow-volumetric incentive exerciser and those who had no IMT. It was posited that it could be because of the structural changes that occur in COPD. These structural changes are found in the central airways, peripheral airways, lung parenchyma, and pulmonary vasculature and such changes are not fully reversible. The results seen on pulmonary function tests in patients with COPD are evidence of over-inflation of the lung (Bullae formation), decreased airflow and abnormalities in gas exchange.^35^

The observation that almost all the cardiovascular parameters (DBP, HR and RPP) showed significant changes in the IS group while the control group showed no significant change also implies that IS brought about the changes. This positive change could be explained by the fact that respiratory modulation is known to be related to cardiovascular modulation and it plays a vital role in blood pressure control. This important interactivity is noted by the generalized alteration that occurs in cardiovascular control in conjunction with respiratory pattern modifications. This relationship is likely related to baroreceptor and chemoreceptor sensitivity interaction and its influence on the mechanisms of blood pressure control.^36^ Comparing the mean changes in the cardiovascular parameters of both groups showed significant differences in all parameters except DBP although the mean changes in DBP was more in the IS group than the control group. May be with longer use of incentive spirometer by patients with T2DM, the comparative mean changes in DBP between the two groups would become significantly different. There is paucity of data on the effect of IS on cardiovascular parameters in patients with T2DM but Ferreira *et al.*^36^ reported that eight weeks of IMT was able to reduce systolic and diastolic blood pressure in patients with hypertension while Mello *et al.*^37^ observed that heart rate and arterial blood pressure in patients with chronic heart failure were not significantly changed in both Control and IMT groups. The differences in the findings of these studies may be due to the different pathologies of the different conditions in the studies.

There was a statistically significant increase of 28.73 m in functional capacity (6MWD) of the IS group post eight weeks of IS compared to a non-statistically significant increase of 7.44 m in the control group who had no IS. This increase of 28.73 m may not be clinically significant as Shoemaker *et al.*^38^ in a systematic review recommended that an increase of 54 m in the 6MWT was considered to be clinically significant. To improve the functional capacity of patients with T2DM so that it is clinically significant, we propose higher intensity IS and longer training period like 12 weeks. Little is known in literature about the effect of IS on the functional capacity of patients with diabetes using 6MWT although the studies by Correa *et al.*^4^ and Kaminski *et al.*^15^ did not show improvement in the functional capacity of patients with diabetes following IMT. This disparity may be because these researchers employed low intensity inspiratory loading (30% of maximal static inspiratory) because various studies where higher intensity IMT were employed showed increases in functional capacity following IMT in patients with COPD, atrial fibrillation and asthma.^34,39,40^

The significant reduction in the fasting blood glucose level (FBS) observed in the IS group and non-significant reduction in the control group imply that IS brought about the difference although the comparison between the mean differences of the two groups showed no significant difference. This is similar to the findings of Correa *et al.*^20^ who reported that high resistance of inspiratory muscle exercise reduces blood glucose levels. It was also posited that IMT improves insulin sensitivity in elderly patients with insulin resistance, hence leading to reduction in blood glucose level.^41^ In another study by Silva *et al.*,^21^ IMT induced a reduction in fasting glucose levels and improved the secretory capacity of pancreatic β cells. These findings are at variance with the study by Ahmad *et al.*,^42^ which observed that IMT with low inspiratory loading failed to demonstrate any significant improvements in blood glucose levels in female patients with type T2DM. This discrepancy could be attributed to the intensity of inspiratory load and the duration of inspiratory exercises. This explanation could be supported by reports from The American College of Sports Medicine and the American Diabetes Association, both of which stated that the reductions in blood glucose levels are related with exercise intensity and duration.^43^ The researchers also explained that the non-significant improvement in blood glucose could be as a result of daily hassles and family stress experienced and reported by some patients in the study group which could have led to elevated plasma glucose levels most of the time. The mechanism is through the direct physiological effects of stress on counter-regulatory hormones, which in turn increases blood glucose.^42^

The finding that there was no significant correlation between most of the pulmonary function parameters (FVC and FEV${}_{1})$ and functional capacity, glycemic control as well as DOD except weak correlation between PEFR and functional capacity as well as DOD may imply that the state of the pulmonary function parameters has no relationship with glycemic control but has minimal relationship with functional capacity and DOD in patients with T2DM. This minimal relationship between the pulmonary function parameters and functional capacity may be due to the fact that the improvements in the pulmonary function parameters within eight weeks of use of incentive spirometer is not sufficient enough to bring about massive improvement in the functional capacity in these patients. If the study had lasted longer, the relationship might have been stronger as greater changes in the pulmonary function parameters may bring about more improvement in the functional capacity in these patients. There is dearth of literature on the relationship between pulmonary function and functional capacity in patients with T2DM. The no significant correlation between the pulmonary function parameters and glycemic control may also be explained by the duration of training with incentive spirometer in these patients. Longer training with incentive spirometer (more than eight weeks) by patients with T2DM may result in significant negative correlation between the pulmonary function parameters and glycemic levels as both parameters were improving significantly with IS training. Shah *et al.*^13^ and Yadav *et al.*^44^ also reported that there is no correlation between pulmonary function and glycemic levels. They argued that glycated Haemoglobin (HbA1c) level is an indicator of glycemic control for a short period of one to two months and that short duration of hyperglycemia was not adequate to influence PFTs. However, Davis *et al.*,^14^ Jamatia *et al.*^45^ and Singh *et al.*^46^ observed a negative correlation between pulmonary function and glycemic levels, indicating that a poor lung function was associated with a poor glycemic control. These authors did not explain the exact pathophysiological mechanism for this association. Concerning DOD, Shah *et al.*,^13^ Yadav *et al.*^44^ and Singh *et al.*^46^ established that there was no relationship between pulmonary function and DOD, hence explaining that DOD did not affect pulmonary function. On the other hand, Davis *et al.*^14^ in their community-based cohort documented that pulmonary function decreased at an average of between 1.1% and 3.1% per year.

## Conclusion

IS brought about significant improvements in the cardiopulmonary function, functional capacity and glycemic control in patients with T2DM. There was no relationship between pulmonary function parameters (FVC and FEV${}_{1})$ and functional capacity although a weak relationship exists between PEFR and functional capacity as well as DOD. There was also no relationship between pulmonary function parameters and glycemic control.

## Relevance of the Study

Based on the findings of this study, it is hereby advocated that IS should be included as a vital aspect of cardiorespiratory physiotherapy in the management of patients with T2DM. More so, it is a cost effective, simple and non-invasive tool that can easily be used by patients.

## Implication for Further Studies

The long-term efficacy of IS training on cardiopulmonary parameters, functional capacity as well as blood glucose levels in patients with T2DM should be researched.

## Conflict of Interest

The authors declare no competing interest, financial or otherwise.

## Funding/Support

There was no grant or financial support from any organization.

## Author Contributions

Happiness A. Aweto and Bosede A. Tella were responsible for conception and design of the study, analysis and interpretation of data, revising the manuscript critically and approval of the final version of the manuscript. Esther O. Obikeh was responsible for acquisition of data as well as drafting and revision of the manuscript.
